# The management of diabetes in indigenous Australians from primary care

**DOI:** 10.1186/1471-2458-7-303

**Published:** 2007-10-25

**Authors:** Mark Thomas, Andrew J Weekes, Merlin C Thomas

**Affiliations:** 1 Dept of Nephrology, Royal Perth Hospital, Box X 2213, Perth, Australia; 2 Servier, Hawthorn, Melbourne, Australia; 3 JDRF/Danielle Alberti Memorial Centre for Diabetes Complications, Baker Medical Research Institute, Melbourne, Australia

## Abstract

**Background:**

Indigenous Australians have high rates of diabetes and its complications. This study examines ethnic differences in the management of patients with type 2 diabetes in Australian primary care.

**Methods:**

Diabetes management and outcomes in Indigenous patients enrolled in the NEFRON study (n = 144) was systematically compared with that in non-Indigenous patients presenting consecutively to the same practitioner (n = 449), and the NEFRON cohort as a whole (n = 3893).

**Results:**

Indigenous Australians with diabetes had high rates of micro- and macrovascular disease. 60% of Indigenous patients had an abnormal albumin to creatinine ratio compared to 33% of non-Indigenous patients (p < 0.01). When compared to non-Indigenous patients, Indigenous patients were more likely to have established macrovascular disease ((adjusted Odds ratio 2.7). This excess in complications was associated with poor glycemic control, with an HbA_1c _≥ 8.0%, observed in 55% of all Indigenous patients, despite the similar frequency use of oral antidiabetic agents and insulin. Smoking was also more common in Indigenous patients (38%*vs *10%, p < 0.01). However, the achievement of LDL and blood pressure targets was the same or better in Indigenous patients.

**Conclusion:**

Although seeing the same doctors and receiving the same medications, glycaemic and smoking cessation targets remain unfulfilled in Indigenous patients. This cross-sectional study confirms Aboriginal ethnicity as a powerful risk factor for microvascular and macrovascular disease, which practitioners should use to identify candidates for intensive multifactorial intervention.

## Background

Indigenous Australians have high rates of diabetes and its complications, including blindness[1], kidney disease[2], and cardiovascular mortality[3]. The reasons for this racial disparity are multifactorial, involving a complex interplay of behavioural, biological, economic and societal factors that vary across health indicators and healthcare settings. One confounding factor is the quality of diabetes care delivered in populations that are culturally and socio-economically disadvantaged [4,5]. Indeed, the fact that minority patients, such as Indigenous Australians, are more likely to receive care at lower-performing facilities in remote and regional sites has biased many comparative studies. Moreover, when controlled for in managed-care settings, ethnicity is not consistently associated with worse processes or outcomes in individuals with type 2 diabetes [4].

The National Evaluation of the Frequency of Renal Impairment cO-existing with NIDDM (NEFRON) study was an large national survey of patients with type 2 diabetes in the Australian primary care setting [6]. The principal aim of this clinic-based study was to determine the frequency of any consultation between any patient with type 2 diabetes and their general practitioner to be complicated by chronic kidney disease, and the factors that influence it's management. In this paper, we examine the management of patients identified by their GP as having an Indigenous ethnic background, and contrast it with the care of non-Indigenous patients consecutively presenting to the same practice, and over the total NEFRON cohort.

## Methods

### Study design

NEFRON was an clinic-based, cluster-stratified survey of patients with type 2 diabetes in the Australian primary care setting. It represents the collaborative effort of Kidney Health Australia, the Baker Heart Research Institute, Servier (Australia) and participating investigators. Data collection and analysis were managed externally by Quintiles SRS and Statistical Revelations, Melbourne. The principal aim of the study was to determine, in the general practice setting, the prevalence of impaired kidney function in individuals with established type 2 diabetes. The Royal Australian College of General Practitioners (RACGP) National Research and Evaluation Ethics Committee approved the study and written informed consent was obtained from all participating patients.

### Sampling and investigator selection

The recruitment of investigators and sample selection are described in detail elsewhere [6,7] and are briefly summarised below. Expressions of interest (EOIs) were invited via a mailing from all (18810) registered GPs across all jurisdictions in Australia in February 2005. EOIs were stratified according to location within each state in a classification derived from Australian Rural, Remote and Metropolitan Area categories. A number of EOIs from each stratum, proportional to the census population (Australian Bureau of Statistics, 2001), were then randomly selected (SPSS V13.0, Quintiles), to make up a total of 500 GP investigators nationally. In the event that a cohort did not achieve target levels of GP registration, re-mailing targeted by sector was employed. In the event of investigator withdrawal from the study, the geographically closest, but not previously selected, EOI was approached as a potential replacement. The final investigators recruited had a mean age, gender distribution, professional qualifications and number of sessions per week that was not significantly different to that recorded for the all registered general practitioners in Australia [6,7].

### Target population/eligibility requirements

During their own routine practice, selected NEFRON investigators were requested to recruit 10–15 consecutively presenting adult patients (18 years of age or older) with established type 2 diabetes, irrespective of reason for visit. Patients were required to give written informed consent in English. Consecutive persons with disabilities that precluded participation in the study, or those declining to participate in the study were not included.

### Survey protocol and procedures

Data collection took place between April and September, 2005. A de-identified case report form was completed for each eligible patient, which captured demographic information including age, gender, ethnicity, history of diabetic complications, medication usage, smoking and relevant family history. Additionally, the results of routine physical examination were provided (height, weight, waist circumference, blood pressure [seated, right arm, diastolic at Korotkov phase V], and data from the most recent blood tests and urinalysis, including serum creatinine, urea and electrolytes, HbA_1c_, fasting glucose and lipids, urinary albumin and creatinine. Tests were repeated at the study visit if no recent (within 3 months) result was available. No attempt was made to standardise results from different laboratories or regions, but rather to document the raw results on which practitioners base their assessment and management.

### Patient stratification

For the purpose of this analysis, the management of diabetic patients identified by their GP as having Aboriginal or Torres Strait Island ethnicity (n = 144), was compared with that received by non-Indigenous patients presenting consecutively to the same practitioner (n = 449), and as well as to that of the total NEFRON cohort.

### Definitions used

For the purposes of analysis, glycaemic control was handled as a categorical variable, with poor glycaemic control denoted by a HbA_1c _above 8.0%, an optimal control denoted by a HbA_1c _below 7.0%. Optimal blood pressure control was denoted by a systolic BP less than 130 mmHg. Optimal lipid control was denoted by achieved IDF treatment targets of an LDL < 2.5 mmol/L, an HDL > 1.0 mmol/L, and triglycerides less than 2.3 mmol/L.

The presence of Chronic Kidney disease (CKD) was defined according to standard Kidney Disease Outcomes Quality Initiative (K/DOQI) guidelines, such that individuals with an estimated glomerular filtration rate (eGFR) of less than 60 ml/min/1.73 m^2 ^or with evidence of kidney damage on urinalysis (e.g. microalbuminuria) were said to have CKD. For primary analyses, the presence of an eGFR of less than 60 ml/min/1.73 m^2 ^was determined as a categorical variable using the MDRD-4 formula, which has been shown to be a reliable tool for the determination of impaired kidney function in Australian patients with type 2 diabetes[8]. Albuminuria was stratified according to Australian and International Diabetes Federation guidelines [9,10], such that women with a urinary ACR of less than 3.5 g/mol and men less than 2.5 g/mol were considered to have Normoalbuminuria. Microalbuminuria was defined in women by a urinary ACR of 3.5 to 35 g/mol and in men by a urinary ACR of 2.5 to 25 g/mol. Macroalbuminuria was defined by a urinary ACR of greater than 35 g/mol in women and greater than 25 g/mol in men. Patients with macro- or microalbuminuria were said to have elevated urinary albumin excretion. Macrovascular disease was defined at the presence of any of cardiovascular disease, cerebrovascular disease or peripheral vascular disease.

### Data handling and Statistical Methods

Continuous data are expressed as their mean ± standard error (SEM). Categorical data are expressed as their frequency (%) in the cohort or subgroup. Analyses for nominal variables consisted of either one-way analysis of variance (ANOVA) for single variables or two-way ANOVA for comparison of three groups. Sub analyses for categorical variables involved Pearson Chi-Square analysis of proportions between independent parameters. The independent influence of Indigenous ethnicity on glycaemic and blood pressure control and on the frequency of diabetic complications, was assessed by multivariate logistic regression analysis, adjusting for differences in baseline parameters including age, gender, duration of diabetes, smoking history, obesity, and the type and intensity of glycaemic, antihypertensive and lipid control. In addition, to correct for potential clustering of responses within practices, confidence intervals and statistical significance were estimated using a sandwich variance-covariance matrix. Multivariate associations are expressed as an adjusted odds ratio [95% confidence interval (CI)].

## Results

### Patient characteristics

**Figure 1:**
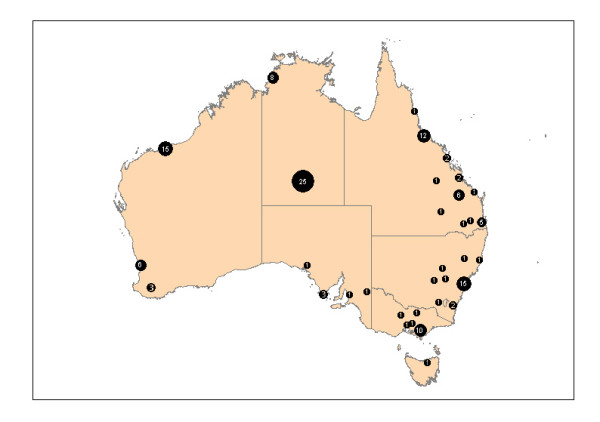
Geographic distribution of Australian practices enrolling Indigenous patients in the NEFRON study.

Data and informed consent were obtained from 3893 adults with type 2 diabetes. The clinical characteristics of this cohort have been previously published[6,7], and are similar to that observed in other Australian studies of patients with type 2 diabetes. Briefly, patient had a mean age of 66 years and a median duration of diagnosed diabetes of six years. Fifty-two percent of all patients were male. 144 patients (3.7%) were identified by their practitioner as Indigenous Australians. These patients came from 51 different practices from urban, rural and remote settings throughout Australia (figure 1). These same practices recorded clinical data on 449 non-Indigenous patients presenting consecutively with Indigenous patients in their practices. The non-indigenous control group was predominantly Caucasian (87%), but included patients of Asian (10%) and other ethnic backgrounds. The clinical characteristics of both these patient groups, and the NEFRON cohort as a whole are detailed in table 1. While all patients had a similar duration of diagnosed diabetes, Indigenous patients were approximately 10 years younger and more often female than other diabetic patients in the same practice or the cohort as a whole (table 1). Indigenous patients also were more likely to have a first-degree relative with diabetes compared to non-Indigenous patients presenting consecutively (68% vs 34%, p < 0.01).

**Table 1:** Clinical characteristics of NEFRON study patients. Univariate p-value compares Indigenous versus non-Indigenous patients from the same practice

	**Indigenous**	**Non-Indigenous but same practice**	**All NEFRON**	**Univariate p-value**
	N = 144	N = 449	N = 3898	
Age (years)	53 ± 1	64 ± 1	66 ± 1	<0.001
Gender (% male)	41% (40–42)	50% (50–51)	52%(51–52)	0.04
Diabetes duration (years)	8 ± 1	8 ± 1	8 ± 1	0.96
Family history of diabetes (%)	64% (58–62)*	37%(33–41)	35%(34–36)	<0.001
Current smoker (%)	38% (30–46)*	9% (6–12)	10%(9–11)	<0.001
Obese BMI(%)	47% (38–56)	52% (47–57)	49% (47–51)	0.31
Waist circumference (cm)	106 ± 2	105 ± 1	104 ± 1	0.48
HbA_1c_(%)	8.9 ± 0.1*	7.4 ± 0.1	7.3 ± 0.1	<0.001
Fasting Glucose (mmol/L)	11.0 ± 0.4*	8.0 ± 0.1	7.9 ± 0.1	<0.001
Metformin (%)	71% (64–78)	69% (65–73)	63% (61–65)	0.60
Sulphonylurea (%)	52% (44–60)	43% (38–48)	45% (43–47)	0.08
Any Insulin (%)	18% (12–24)	14% (11–17)	13% (12–15)	0.21
Antihypertensive (%)	66% (58–76)	70% (66–74)	71% (70–72)	0.47
RAS blockade (%)	76% (69–83)	69% (65–73)	71% (70–72)	0.08
Number Antihypertensives (n)	1.1 ± 0.1*	1.3 ± 0.1	1.4 ± 0.1	0.03
Blood Pressure (mmHg)	132/80	134/78	134/77	0.24/0.05
Lipid lowering therapy (%)	52%(44–60)	59%(54–64)	64%(62–66)	0.17
LDL cholesterol (mmol/L)	2.5 ± 0.1	2.6 ± 0.1	2.5 ± 0.1	0.80
HDL cholesterol (mmol/L)	1.1 ± 0.1*	1.2 ± 0.1	1.3 ± 0.1	<0.001
Triglycerides (mmol/L)	2.7 ± 0.1*	2.0 ± 0.1	2.0 ± 0.1	<0.001
Normoalbuminuria (%)	41% (33–49)*	67% (63–71)	65%(63–67)	<0.001
Microalbuminuria (%)	36% (28–44)*	27% (23–31)	27%(26–28)	<0.001
Macroalbuminuria (%)	23% (16–30)*	6%(4–8)	7%(6–8)	<0.001
eGFR < 60 ml/min/1.73 m^2^	21%(14–28)	19%(14–24)	23%(21–25)	0.50
Family history of CKD (%)	38% (30–46)*	8%(5–11)	7%(6–8)	<0.001
Macrovascular disease (%)	35%(27–43)	27%(23–31)	31%(30–32)	0.08
Cardiovascular disease (%)	25%(18–32)	18%(14–22)	23%(22–24)	0.10
Family history of early CVD (%)	43%(34–51)	14%(10–18)	15%(14–16)	<0.001

( * p < 0.01; adjusted for age, gender and duration of diabetes)

### Glycaemic control in Indigenous patients

Metabolic control was significantly worse in Indigenous patients than other diabetic patients in the same practice or the NEFRON cohort as a whole. While 48% of all NEFRON patients achieved HbA_1c _targets of < 7.0%, these targets were achieved in only 24% of Indigenous patients. Moreover, Indigenous Australians were more likely to have an HbA_1c _≥ 8.0% (55%) when compared to Caucasian (23%) or Asian patients in the NEFRON survey (23%). This excess persisted after adjusting for other risk factors for poor glycaemic control (adjusted odds ratio 2.8; 95% CI 2.0 – 4.3). In addition, the use of oral antidiabetic agents, alone or in combination, and insulin was similar, in all ethnic groups (table 1).

When asked to categorise their patient's glycaemic control, on the basis of current results, 52% of NEFRON patients were perceived by their GP as having 'optimal control', including 28% of Indigenous patients. In both Indigenous and non-Indigenous patients, this categorisation closely concurred with a target HbA_1c _of 7.0% (kappa statistic for agreement = 0.76), suggesting that practitioners were not complacent about poor glycaemic control in their Indigenous patients.

### Blood pressure control in Indigenous patients

While mean blood pressure levels were similar in the NEFRON cohort regardless of ethnicity (table 1), more Indigenous patients achieved treatment targets of systolic BP < 130 mmHg (44%) when compared to 33% in the NEFRON cohort (p = 0.01). This was partly explained by the fact that Indigenous patient were generally younger, and had a shorter duration of diabetes (table 1). After adjusting for these variables, Indigenous patients were equally likely to achieve treatment targets (adjusted Odds ratio 1.1; 95% CI 0.8 – 1.4). At the same time, GPs identified the BP to be 'optimally controlled' in 66% of Indigenous patients, compared to 65% in the cohort as a whole. The percentage of subjects prescribed agents that block the RAS were also similar to the cohort as a whole. However, Indigenous patients were less likely to be receiving multiple antihypertensive agents (27%) compared to other diabetic patients presenting at the same centre (40%) or in the cohort as a whole (43%).

### Lipid levels in Indigenous patients

Total cholesterol levels were generally similar in Indigenous patients when compared to diabetic patients in the same practice or to the NEFRON cohort as a whole (table 1). 53% achieved treatment targets of LDL < 2.5 mmol/L compared to 54% in the NEFRON cohort, and this identity persisted after adjusting for baseline differences (adjusted Odds ratio 1.1; 95% CI 0.7 – 1.6). A similar number of both Indigenous and non-Indigenous patients also received lipid-lowering therapies. However, Indigenous patients were more likely to have a low HDL (<1.0 mmol; 39% vs 21% in non-Indigenous patients seen consecutively at the same practice and 16% in the cohort as a whole; adjusted Odds ratio 4.0; 95% CI 2.6 – 6.0). In addition 52% of all Indigenous patients had elevated triglycerides (≥ 2.3 mmol/L) compared to 26% of the NEFRON cohort as a whole (adjusted Odds ratio Odds ratio 2.7; 95% CI 1.8 – 3.6).

### Microvascular Disease in Indigenous patients

**Figure 2:**
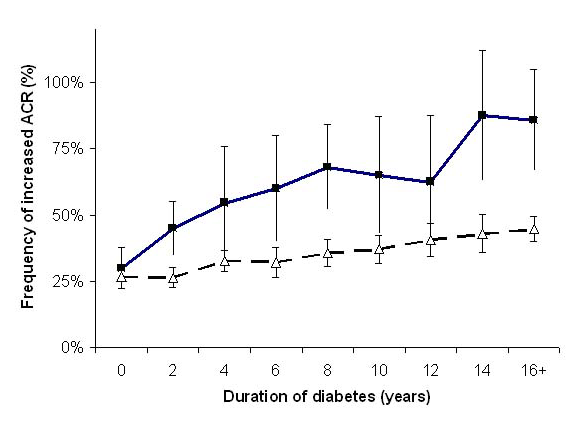
The frequency of an elevated urinary ACR (± 95% CI), stratified according to duration of diabetes. Dark squares denote Indigenous patients, white triangles denotes the total NEFRON cohort.

Indigenous patients had high rates of CKD, despite the same median duration of diabetes. In particular, 23% of Indigenous patients with type 2 diabetes had a urinary ACR in the macroalbuminuric range, compared to 6% of non-Indigenous patients in the same practice or 7% in the NEFRON cohort as a whole. Overall, after adjusting for baseline differences, Indigenous patients presenting to their GP were significantly more likely to have an increased urinary albumin excretion (adjusted Odds ratio 5.2; 95% CI 3.6 – 4.6). This excess in renal disease was clearly familial, as 38% of Indigenous patients had first-degree relative with CKD, compared to less than 8% of non-Indigenous patients presenting consecutively. However, this excess of Indigenous patients with had an elevated urinary ACR could not be explained by higher albumin excretion rates more generally in Indigenous individuals, as 28% of non-Indigenous patients with newly-diagnosed diabetes (duration less than one year) had an abnormal ACR compared to 32% of Indigenous patients with newly diagnosed diabetes (figure 2).

**Figure 3:**
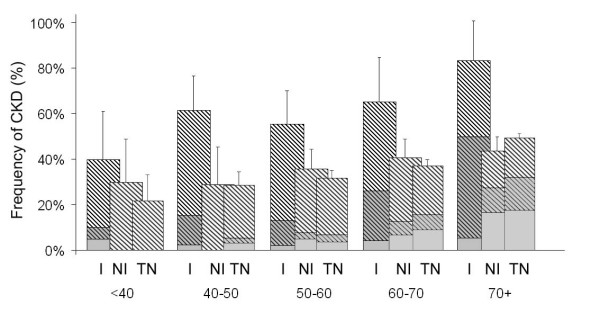
The unadjusted frequency of (a) CKD, stratified according to age and ethnicity. Crosshatch denotes patients with an elevated ACR, shading denotes patients with an eGFR < 60 mol/min/1.73 m^2^, I = Indigenous, NI = Non-Indigenous, TN = total NEFRON cohort. Error bar denotes 95% confidence interval for CKD.

Twenty-one percent of patients in the NEFRON survey had a reduced eGFR (<60 ml/min/1.73 m^2^), regardless of ethnicity. This was partly due to the large numbers of elderly Caucasian patients with a reduced eGFR seen in Australian medical practice and enrolled in the NEFRON survey. However, after adjusting for baseline differences, Indigenous patients presenting to their GP were significantly more likely to have a reduce eGFR (adjusted Odds ratio 2.4; 95% CI 1.4 – 3.9, figure 3). Retinopathy was also more commonly identified in Indigenous patients (16% vs 10%, adjusted Odds ratio 2.2; 95% CI 1.2 – 3.6), as previously reported [6,7].

### Macrovascular disease in Indigenous patients

**Figure 4:**
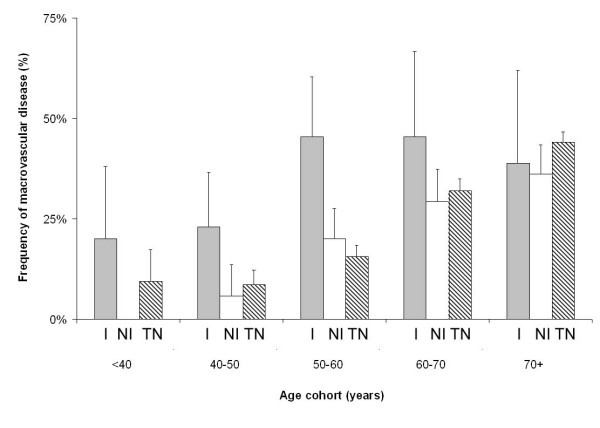
The unadjusted frequency of macrovascular disease, stratified according to age and ethnicity. I = Indigenous, NI = Non-Indigenous, TN = total NEFRON cohort. Error bar denotes 95% confidence interval

Thirty-one percent of all patients presenting to their GP in the NEFRON study had symptomatic macrovascular disease, including 23% with cardiovascular disease (table 1). Overall, the frequency of macrovascular disease was marginally more common in Indigenous patients compared other patients from the same practices. This rate was not significantly different from the NEFRON cohort as a whole (table 1). However, after adjusting for baseline differences, Indigenous patients were significantly more likely to have established macrovascular disease (adjusted Odds ratio 2.7; 95% CI 1.8 – 4.2, figure 4). This was not attributable to the excess of CKD in this population, as a high rate of macrovascular disease was seen in Indigenous patients with and without CKD (35% vs 34%, p = NS). However, 43% of Indigenous patients had first-degree relative with cardiovascular disease diagnosed before the age of 50, compared to less than 15% of non-Indigenous patients presenting consecutively. Indigenous patients were also more likely to be current smokers. Despite their increased cardiovascular risk, aspirin use in Indigenous patients was similar to the NEFRON population as a whole (~51%).

## Discussion and conclusion

Despite advances in diabetes care, the clinical outcomes of Indigenous Australians with type 2 diabetes in general practice remain poor. While ethnic differences in the quality of diabetes care partly contributes to disparity in diabetic complications, even when compared to non-Indigenous patients consecutively attending the same practitioner and receiving a comparable pattern of care, Aboriginal patients have worse glycemic control, and more micro- and macrovascular complications. These findings are consistent with smaller community-based studies[11] and collected regional data from remote and rural settings [12-15], where HbA_1c _levels are typically 1% higher in Indigenous patients, and high rates of complications are observed. However, these data represent the first national survey including large numbers of Indigenous patients and adjusting for centre effects, to characterise this disparity.

The National Evaluation of the Frequency of Renal Impairment cO-existing with NIDDM (NEFRON) study was an clinic-based, cluster-stratified survey of patients with type 2 diabetes from across the Australian primary care setting. It was not designed to be representative of all patients with type 2 diabetes, and clearly excludes patients outside of the mainstream of primary care (e.g. Aboriginal Medical Services, Tertiary Care etc). Data collected as part of NEFRON also relied on the GP's assessment of each patient, which may be inaccurate with regards to assigned ethnicity or other factors associated with diabetes management. As a clinic-based incident-driven study it has a number of limitations, being inherently (and deliberately) biased towards the kind of patients that regularly attend their practitioner, and the non-standardised laboratory results on which they base their daily management. Nonetheless, from the practitioner's perspective, the likelihood that any routine GP-patient encounter will be accompanied by specific complications of diabetes, and the opportunities for improvement within the context of established practice may be better estimated by a clinic-based survey, when compared to cross-sectional population studies including individuals who are never seen by practitioners, and data not available to practitioners. However, as Indigenous patients are less likely to seek care from a General Practitioner (Family Physician), there could be a bias towards more unwell subjects in that group, including those with a history of cardiovascular or kidney complications. Equally non-compliant individuals who carry a greater burden of complications, may be less likely to be represented in an encounter-driven survey. Nonetheless, the percentage of NEFRON patients with an Indigenous background (3.7%) was similar to that observed in other recent cross-sectional studies, including the DEMAND and the AusDiab surveys (3%) where ethnicity was self-identified (Robert Atkins, personal communication).

Although every effort was undertaken to ensure a representative distribution of practices through random selection of investigators[6,7], initial recruitment in the NEFRON study was also based on interest in undertaking the study. It is also possible that these clinics might therefore not be an accurate representation of primary care services accessed by Indigenous Australians with diabetes. Consequently some selection bias in relation to participating investigators and subsequently enrolled diabetic patients, cannot be ruled out. Nonetheless, the practice and practitioner characteristics of the NEFRON study investigators, were similar to that recorded for Australian general practice as a whole, and on the whole represent the 10,000 encounters between patients with type 2 diabetes and their GP that occur every working day in Australia.

While the NEFRON study shows that ethnic disparities persist even within the same practice, this survey did not include individual data on socio-economic, educational, cultural and other factors known to impact diabetes management. Poverty, lack of education and other factors associated with socio-economic disadvantage are strongly linked to adverse health outcomes, independent to ethnicity. However, Indigenous Australian's carry a much greater burden. As seen in other minority populations worldwide[16], socio-economic disadvantage is strongly correlated with the higher prevalence of chronic disease seen in Aboriginal peoples [17,18]. Data from the community-based Fremantle Diabetes study suggest that major disparities in income, housing and eduction may underlie many of the ethnic differences in diabetes care[11]. Similarly, issues regarding acceptability, uptake and compliance have not been addressed in the NEFRON study. However, recent data suggest that uniform access to and quality of care can result reduce process or outcome disparities in diabetes care[4]. While such data have not been confirmed in Indigenous Australians, even after adjusting for practice differences by analysing consecutive patients, key areas of unmet need in Indigenous patients with type 2 diabetes attending their GP may be clearly demonstrated.

Albuminuria was more common in Indigenous patients in this survey, where 23% of patients with type 2 diabetes had an ACR in the macroalbuminuric range, compared to 6% of non-indigenous individuals. However, the NEFRON study was limited by restricting classification of the UAE to the most recent ACR for any individual patient, Since there is known to be significant day-to-day variability in albumin excretion, ideally, at least two of three collections in a 3- to 6-month period should show elevated levels before a patient is classified as having an abnormal albumin excretion. Nevertheless, the NEFRON data are consistent with findings from the AusDiab study, which employed three collections and found that 34% of individuals with diabetes had an elevated UAE[19]. Although there is strong familial clustering of CKD in Indigenous patients, some of the observed excess in CKD may be attributable to modifiable differences in health care delivery, including glycaemic control and smoking cessation [20,21]. There were no clear differences in mean blood pressure control or the initiation or use of antihypertensive therapy. Indeed, blood pressure targets were more often achieved in Indigenous patients, consistent with previous reports [11]. Similarly, the measures of obesity were similar in Indigenous and non-Indigenous cohorts, although such parameters have not been validated in Indigenous Australians.

It has been argued that micro- and macro-albuminuria in Aboriginal people is common in the absence of diabetes. Indeed, it has been suggested that the 'normal' range for microalbuminuria and other variables may be different in Indigenous and non-indigenous groups. Nonetheless, from a practitioner's perspective, the finding of CKD in Indigenous patient with diabetes is a potent risk factor for adverse outcomes, and should not be dismissed as a racial difference. While a date of diagnosis of diabetes is not the same as the onset of diabetes (which is likely to have been at least 5–10 years earlier), our data suggests that similar numbers of Indigenous and non-Indigenous patients with recently diagnosed diabetes had an abnormal ACR, defined according to conventional guidelines. However, at later time-points, albuminuria was more common, potentially reflecting accelerated microvascular disease in Indigenous patients.

Although microvascular disease was more common in Indigenous patients, the frequency of patients with macrovascular disease or impaired kidney function was overall very similar to that observed in non-Indigenous patients. Such data may suggest a survival bias, whereby Indigenous patients who have died or attended tertiary hospitals because of more severe complications (e.g. ESRD, CHF), would not have been included in the NEFRON study. Indeed, Indigenous patients with type 2 diabetes were on average 11 years younger than non-Indigenous probands, making cardiovascular events and ESRD appear less common, though at the same time, after age adjustment, inordinately high (figure 4). Moreover, 43% of Indigenous patients with type 2 diabetes had a first degree relative with cardiovascular disease diagnosed before the age of fifty.

There are many potential contributors to impaired glycaemic control in Indigenous patients. Although from the NEFRON survey it is apparently that patients are receiving the same combinations of the same antidiabetic drugs, and the targets for optimal glycaemic control are the same, few Indigenous patients are reaching these targets. Compliance with prescribed antidiabetic therapies, lifestyle and dietary modifications are likely contributors to this disparity, possibly reflecting issues surrounding the frequency, accessibility, affordability, and cultural sensitivity of diabetic care. Genetic predisposition, dyslipidemia, chronic inflammation and reduced 'endowment' of pancreatic islets (much as nephrons) in low-birth weight babies, may also make diabetes more difficult to treat. It is interesting to note that racial differences in metabolic control diminished with the duration of diabetes. Whether this is a lead-time effect of late diagnosis of diabetes, or the loss of recalcitrant patients to co-morbid disease remains to be established by longitudinal studies. However, it is plausible that at the time of presentation Indigenous patients have more advanced β-cell depletion and higher levels of insulin resistance, making them less sensitive to standard oral antidiabetic therapies, in a similar way described in Caucasian populations with advanced disease[22]. This has led to the suggestion that both groups would potentially benefit from insulin therapy[11], a treatment approach which remains underutilised in the general practice management of type 2 diabetes.

The NEFRON study was designed to examine factors that influence the management of diabetes in the context of GP-patient encounter. As such, it does not address the pivotal socio-economic determinates of health, including income and social status; social support networks; education and literacy; employment and working conditions; social environments; physical environments; and housing; which also contribute to high rates of diabetes and its complications. It is clear that Indigenous Australians Islanders will not achieve equal health outcomes until their economic, educational and social disadvantages have been eliminated[23]. Nevertheless, while social disadvantage continues, the NEFRON study also demonstrates key ethnic disparities in Australian diabetes care. The aim of the national health care funding system is to give universal access to health care. Yet while seeing the same doctors and receiving the same medications, treatment targets remain unfulfilled in Indigenous patients. More importantly, intensification of management, which should follow the identification of risk, is seldom applied. This cross-sectional study confirms Aboriginal ethnicity as a powerful risk factor for microvascular and macrovascular disease, which should be used by practitioners to identify candidates for more intensive multifactorial intervention.

## Competing interests

The author(s) declares that there are no competing interests.

## Authors' contributions

MCT and AW carried out the NEFRON study, as a collaboration between the Baker Heart Research Institute, Kidney Health Australia and Servier Australia. MT an MEC participated in data analysis and preparation of this manuscript. All authors read and approved the final manuscript.

## Pre-publication history

The pre-publication history for this paper can be accessed here:


